# Subclinical atrial fibrillation detection with a floating atrial sensing dipole in single lead implantable cardioverter‐defibrillator systems: Results of the SENSE trial

**DOI:** 10.1111/jce.14081

**Published:** 2019-08-05

**Authors:** George Thomas, Daniel Y. Choi, Harish Doppalapudi, Mark Richards, Sei Iwai, Emile G. Daoud, Mahmoud Houmsse, Arvindh N. Kanagasundram, Sumeet K. Mainigi, Steven A. Lubitz, Jim W. Cheung

**Affiliations:** ^1^ Division of Cardiology, Weill Cornell Medicine NewYork‐Presbyterian Hospital New York New York; ^2^ Division of Cardiology University of Alabama at Birmingham Birmingham Alabama; ^3^ Northwest Ohio Cardiology Consultants Toledo Ohio; ^4^ Division of Cardiology Westchester Medical Center Valhalla New York; ^5^ Division of Cardiovascular Medicine The Ohio State University Medical Center Columbus Ohio; ^6^ Division of Cardiology Vanderbilt University Nashville Tennessee; ^7^ Department of Cardiology and Electrophysiology Albert Einstein Medical Center Philadelphia Pennsylvania; ^8^ Division of Cardiology Massachusetts General Hospital Boston Massachusetts

**Keywords:** implantable cardioverter‐defibrillator, remote monitoring, subclinical atrial fibrillation

## Abstract

**Introduction:**

Subclinical atrial fibrillation (AF), in the form of cardiac implantable device‐detected atrial high rate episodes (AHREs), has been associated with increased thromboembolism. An implantable cardioverter‐defibrillator (ICD) lead with a floating atrial dipole may permit a single lead (DX) ICD system to detect AHREs. We sought to assess the utility of the DX ICD system for subclinical AF detection in patients, with a prospective multicenter, cohort‐controlled trial.

**Methods and Results:**

One hundred fifty patients without prior history of AF (age 59 ± 13 years; 108 [72%] male) were enrolled into the DX cohort and implanted with a Biotronik DX ICD system at eight centers. Age‐, sex‐, and left ventricular ejection fraction‐matched single‐ and dual‐chamber ICD cohorts were derived from a Cornell database and from the IMPACT trial, respectively. The primary endpoint were AHRE detection at 12 months. During median 12 months follow‐up, AHREs were detected in 19 (13%) patients in the DX, 8 (5.3%) in the single‐chamber, and 19 (13%) in the dual‐chamber cohorts. The rate of AHRE detection was significantly higher in the DX cohort compared to the single‐chamber cohort (*P* = .026), but not significantly different compared to the dual‐chamber cohort. There were no inappropriate ICD therapies in the DX cohort. At 12 months, only 3.0% of patients in the DX cohort had sensed atrial amplitudes less than 1.0 mV.

**Conclusion:**

Use of a DX ICD lead allows subclinical AF detection with a single lead DX system that is superior to that of a conventional single‐chamber ICD system.

**Abbreviations**
AF: atrial fibrillation
**AHRE**: atrial high rate episode
BPM: beats per minute
EGM: electrogram
ICD: implantable cardioverter‐defibrillator
LVEF: left ventricular ejection fraction
RV: right ventricle

## 1 INTRODUCTION

Implantable cardioverter‐defibrillators (ICDs) are a cornerstone therapy for the prevention of sudden cardiac death by treating life‐threatening ventricular arrhythmias. In randomized trials establishing the benefit of ICDs for the sudden cardiac death prevention, the majority of patients received single‐chamber ICDs.^1^ However, according to data from the National Cardiovascular Data Registry (NCDR) and other international registries, more patients receive dual‐chamber ICDs than single‐chamber ICDs for primary prevention.^1^, ^2^, ^3^ This remains the case despite the fact that 60% of patients who receive dual‐chamber ICDs for primary prevention of sudden cardiac death do not have an atrial pacing indication.^4^ Multiple studies have shown that implantation of dual‐chamber ICDs is associated with higher rates of procedural complications, in‐hospital mortality, and need for earlier generator change.^1^, ^3^, ^4^

The diagnostic capabilities offered by the presence of atrial intracardiac electrograms (EGMs), however, can provide benefits. Some studies comparing outcomes of dual‐ vs single‐chamber sensing show reduction of inappropriate ICD therapies with dual‐chamber sensing due to supraventricular tachycardia discrimination, although this is not a consistent finding.^5^, ^6^, ^7^ Adoption of ICD programming strategies, utilizing higher rates and longer durations for tachycardia detection, have been more beneficial in reducing inappropriate therapies.^8^, ^9^ Nonetheless, atrial EGMs allow physicians to accurately diagnose previously undetected atrial and ventricular arrhythmias in patients with ICDs.^10^ Specifically, subclinical atrial fibrillation (AF), manifest as device‐detected atrial high rate episodes (AHREs), is associated with increased risk of stroke and mortality.^11^, ^12^, ^13^

A single lead ICD system with a floating atrial dipole may allow AHRE detection without the need for implantation of an additional atrial lead. The single lead DX ICD system (Biotronik, Berlin, Germany) utilizes a proprietary ICD lead with a floating atrial dipole 15 to 17 cm from the tip of the ICD lead and an ICD generator with specialized amplification and filtering to allow the atrial signals to be increased four‐fold, while minimizing far‐field R‐wave sensing. In this multicenter prospective trial, we sought to assess the utility of the single lead DX ICD system for identifying AHREs in patients with no prior history of AF and comparing its rates of AHRE detection with patients in single‐ and dual‐chamber ICD cohorts.

## 2 METHODS

### 2.1 SENSE prospective study population

The SENSE trial (Clinicaltrials.gov identifier NCT02186704) was initiated by study investigators, sponsored by Weill Cornell Medicine and funded by Biotronik, USA. The SENSE trial was a prospective, cohort‐controlled, and eight‐center study comprised of patients who met standard indications for a primary or secondary prevention ICD. Enrollment was conducted from 29 July 2014 to 21 March 2017. All prospective study patients underwent implantation of a Biotronik DX ICD system (Biotronik) with a DX ICD generator (Ilesto 7 VR‐T DX, Iforia 7 VR‐T DX, Itrevia 7 VR‐T DX, Inventra 7 VR‐T DX, or Iperia 7 VR‐T DX) and a DX ICD lead (Linoxsmart S DX 65/15 or Linoxsmart S DX 65/17). Patients were enrolled in the study either before ICD implant or within 30 days after ICD implant. Patients were excluded for: presence of atrial pacing indication, prior history of AF or atrial flutter, AHRE detection before study enrollment, atrial sensing less than 2 mV via the DX ICD system (not the pacing system analyzer), inability to comply with remote monitoring, or life expectancy of less than 1 year. The institutional review board at each center approved the study protocol and written informed consent was obtained from each patient.

### 2.2 Control population

Two historical control cohorts were utilized for the study: a single‐chamber ICD cohort and a dual‐chamber ICD cohort. The single‐chamber ICD cohort was derived from a retrospective database of patients who underwent single‐chamber ICD implantation at Weill Cornell Medicine. Patients with a prior history of AF or atrial flutter or follow‐up less than 1 year were excluded. The dual‐chamber ICD cohort was derived from a subset of patients of the previously conducted IMPACT trial (Clinicaltrials.gov identifier NCT0559988).^14^ The IMPACT trial was a study of 2718 patients with Biotronik dual‐chamber ICDs and biventricular ICDs who were randomized to anticoagulation treatment based on remote rhythm monitoring or office‐based follow‐up for the detection of AHREs. The dual‐chamber control group for this study was derived from the subset of patients who had dual‐chamber ICDs and had no prior history of AF or atrial flutter. Based on age‐, sex‐ and left ventricular ejection fraction (LVEF)‐matching to the prospective SENSE study patient group, 150 out of 184 patients in the single‐chamber ICD database and 150 out of 1740 patients in the IMPACT study subgroup were selected to create the final control cohorts. All control group matching was performed with blinding to the presence of AF or AHRE detection.

### 2.3 ICD programming

All SENSE prospective study patients (DX cohort) had AHRE detection programming based on a Holter AT trigger mode with an intervention rate of 36 out of 48 to start and 20 out of 24 to end an episode, with a beat entry/exit count of 200 beats per minute (bpm) with EGMs for monitored episodes on. All other ICD programming parameters were made at the discretion of the individual operators. All the IMPACT trial dual‐chamber ICD control cohort patients also had a Holter AT trigger mode with an intervention rate of 36 out of 48 to start and 20 out of 24 to end an episode, with a beat entry/exit count of 200 bpm with EGM for monitored episodes on. All other ICD programming parameters were made at the discretion of the individual operators. Single‐chamber cohort patients had ICD programming parameters made at the discretion of the individual operators.

### 2.4 Data collection

**Figure 1:**
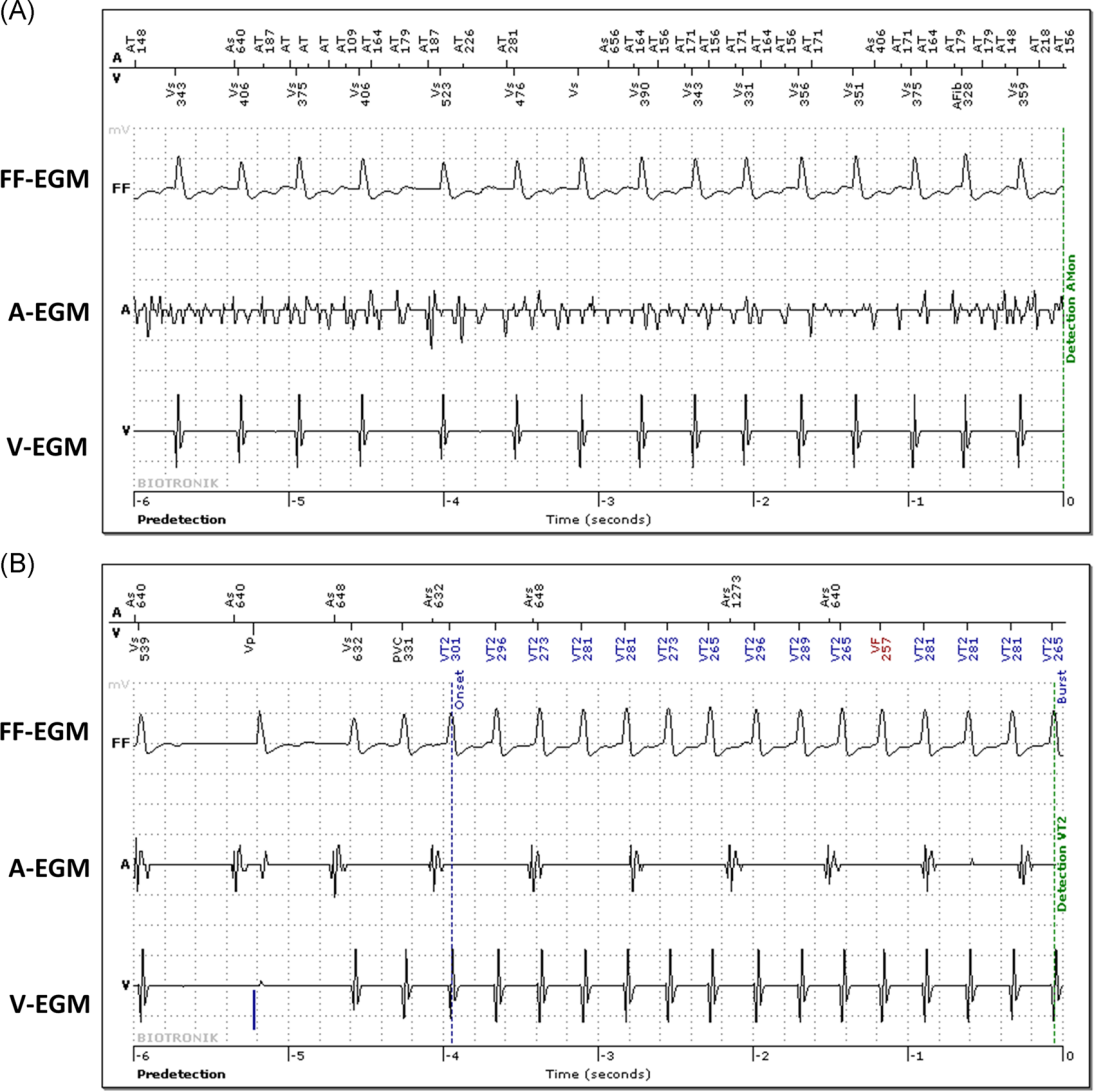
Atrial and ventricular arrhythmia detection in a patient with a DX ICD system. A, Newly detected atrial fibrillation is shown with rapid, disorganized atrial activity (AEGM), and rapid ventricular response (VEGM). This episode lasted 8 minutes. B, Ventricular tachycardia is shown here in the same patient which was subsequently terminated with anti‐tachycardia pacing. Clear dissociation between the atrial (AEGM) and ventricular (VEGM) electrograms is seen. AEGM, atrial electrogram; FFEGM, far‐field electrogram; VEGM, ventricular electrogram

DX cohort patients were followed for 12 months, with remote interrogations every 3 months and in‐office visits at 1‐, 6‐ and 12‐ month post‐ICD implantation. Dual‐chamber cohort patients were followed with in‐office visits at 3‐ to 6‐month intervals and daily remote monitoring transmissions. AHREs were defined as atrial tachyarrhythmias with an atrial rate greater than 200 bpm lasting for greater than 30 seconds. For the single‐chamber cohort, AHREs was defined as any clinical atrial flutter or fibrillation detected via in‐office interrogation, remote interrogation, or electrocardiogram. EGM for AHRE detections in the DX cohort and dual‐chamber cohort and ventricular high rate detections in the single‐chamber cohort were adjudicated by two investigators (JC and GT), independently (Figure 1).

### 2.5 Endpoints

The primary endpoint was AHRE detection lasting greater than 30 seconds by 12 months as per intention‐to‐treat analysis. This study was designed to compare AHRE detection between the DX cohort and the dual‐chamber IMPACT control cohort and to compare AHRE detection between the DX cohort and the single‐chamber control cohort. Secondary endpoints included: AHRE detection lasting greater than 6 minutes, time to detection of first AHRE, appropriate and inappropriate ICD therapies, adverse device events, and mortality. Additionally, device performance parameters such as atrial sensing, ventricular sensing, and ventricular pacing threshold in the DX cohort were assessed.

### 2.6 Statistical analysis

Categorical variables were expressed as the number of observations and the proportion of patients. Continuous variables were expressed as mean ± standard deviation or median and interquartile range based on normality of distribution. Baseline characteristics were compared between the three arms using the *χ* ^2^ test for categorical variables and one‐way analysis of variance with post hoc Scheffe test for continuous variables. Proportions of patients with AHRE detection were compared between the DX cohort and the single‐chamber cohort and between the DX cohort and the dual‐chamber cohort using pairwise the *χ* ^2^ test. Hazard curves for AHRE detection were created with the Kaplan‐Meier method and compared using the log‐rank statistic. To identify predictors of AHRE detection, a multivariable Cox proportional hazards regression model was created by including covariates that had univariate significance (*P* < .10). *P* < .05 was considered statistically significant. Statistical analysis was performed with SPSS version 24.0 (IBM Corp, Armonk, NY).

## 3 RESULTS

### 3.1 Patient characteristics

The prospective DX cohort consisted of 157 patients consented, 7 patients excluded for not meeting enrollment criteria, and 150 patients included in the final analysis. Of the 150 patients, 132 (88%) completed 12 months follow‐up. During follow‐up, 7 (4.7%) patients died, 6 (4%) patients withdrew consent, 3 (2%) patients had ICD system explant without DX system reimplant, and 2 (1.3%) patients were lost to follow‐up. In the single‐chamber cohort, the prevalence of device manufacturer was Medtronic (Mounds View, MN) 64 (43%), Boston Scientific (St Paul, MN) 40 (27%), Abbott (Lake Bluff, IL) 41 (27%), and Biotronik (Berlin, Germany) 5 (3%). In the DX cohort, the number of days from implant to enrollment was median 1 (interquartile range [IQR]: 0, 10.8) and mean 5.8 days. In the dual‐chamber cohort, the number of days from implant to enrollment was median 22.5 (IQR: 8, 56.8) and mean 92.5 days. The baseline characteristics of the three cohorts are listed in Table 1.

**Table 1:** Baseline characteristics

Characteristic	DX cohort (n = 150)	Dual‐chamber cohort (n = 150)	Single‐chamber cohort (n = 150)	*P*
Age, mean ± SD, y	59 ± 13	59 ± 13	54 ± 17	.002*, **
Male, n (%)	108 (72)	108 (72)	108 (72)	1.000
LVEF, mean ± SD	33 ± 17	33 ± 16	31 ± 16	.453
Primary prevention, n (%)	132 (88)	134 (89)	122 (81)	.098
CHF, n (%)	95 (63)	127 (85)	131 (87)	<.001*, ^†^
Hypertension, n (%)	108 (72)	121 (81)	89 (58)	<.001*, **
Diabetes, n (%)	48 (32)	46 (31)	50 (33)	.885
CAD, n (%)	75 (50)	95 (63)	87 (58)	.063
CVA/TIA, n (%)	18 (12)	10 (7)	5 (3)	.015*
CHA2DS2‐VASc score, mean ± SD	2.1 ± 1.2	3.3 ± 1.4	3.1 ± 1.8	<.001*, ^†^
β‐Blocker, n (%)	133 (89)	145 (97)	134 (89)	.022**, ^†^
ACE‐inhibitor/ARB, n (%)	88 (59)	57 (38)	113 (75)	<.001*, **, ^†^
Digoxin, n (%)	12 (8)	11 (7)	7 (5)	.472
Aspirin, n (%)	97 (65)	60 (40)	105 (70)	<.001**, ^†^
Anticoagulant, n (%)	19 (13)	2 (1)	15 (10)	.001**, ^†^

Abbreviations: ACE, angiotensin‐converting enzyme; ARB, angiotensin receptor blockers; CAD, coronary artery disease; CHF, congestive heart failure; CVA, cerebrovascular accident; TIA, transient ischemic attack.

* DX arm vs single‐chamber arm *P* < .05.

** Single‐chamber arm vs dual‐chamber arm *P* < .05.

^†^ DX arm vs dual‐chamber arm *P* < .05.

### 3.2 Subclinical AF detection

**Figure 2:**
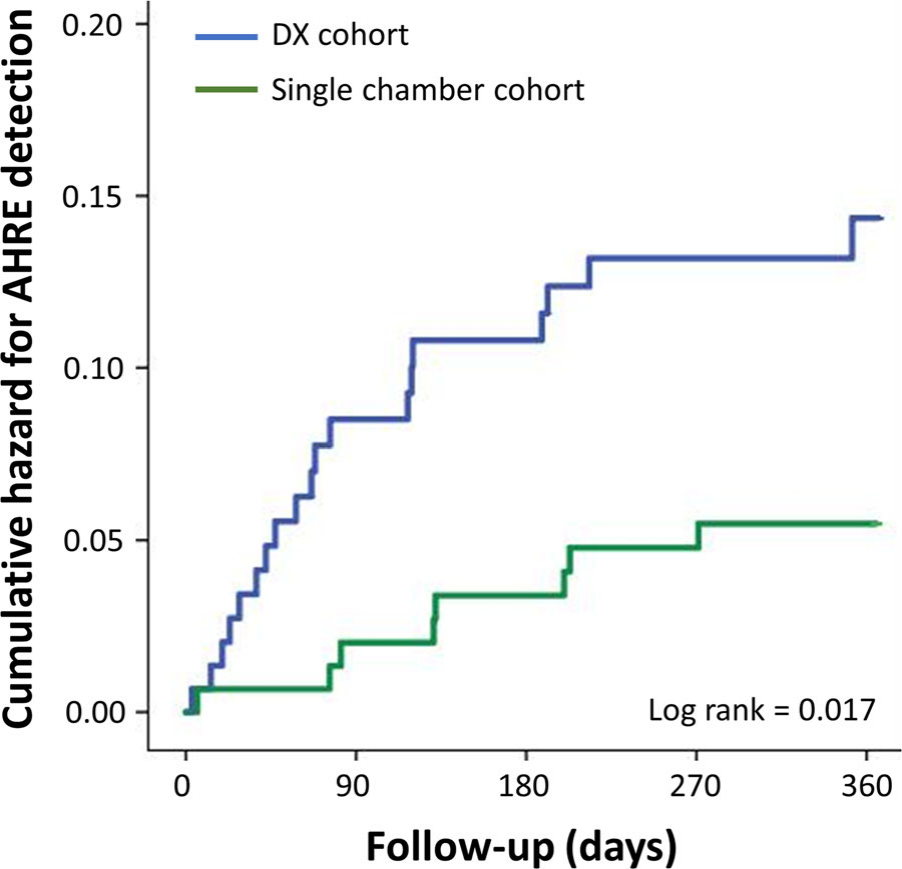
Comparison of hazard curves of atrial high rate episode detection in the DX cohort and the single‐chamber cohort. AHRE, atrial high rate episode

**Figure 3:**
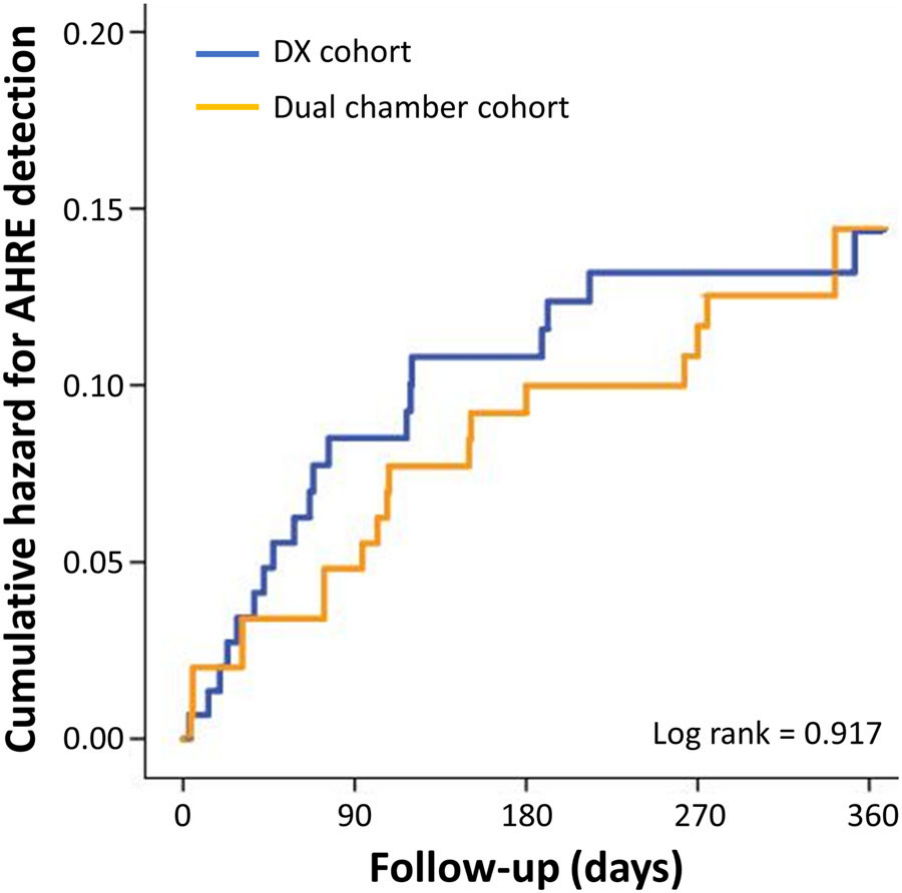
Comparison of hazard curves of atrial high rate episode detection in the DX cohort and the dual‐chamber cohort. AHRE, atrial high rate episode

At 12 months, newly detected AHREs greater than 30 seconds duration were found in 19 (13%) patients in the DX cohort, 19 (13%) in the dual‐chamber cohort, and 8 (5.3%) patients in the single‐chamber cohort. The proportion of patients with subclinical AF detection was significantly higher in the DX cohort when compared to the single‐chamber cohort (*P* = .026). There was no difference in the rate of AHRE detection between the DX cohort and the dual‐chamber cohort (*P* = 1.00). Kaplan‐Meier estimates of time to first AHRE detection revealed increased AHRE detection in the DX cohort compared to the single‐chamber cohort (log‐rank *P* = .017; Figure 2). On multivariable analysis of the combined DX and single‐chamber cohorts, use of the DX system was independently associated with AHRE detection (adjusted HR: 2.40; 95% CI: 1.05‐5.48; *P* = .038). On survival analysis, there was no difference in AHRE detection between the DX cohort and the dual‐chamber cohort (log‐rank *P* = .917; Figure 3). At 12 months, AHREs greater than 6 minutes duration were detected in 16 (11%) patients in the DX cohort and 15 (10%) in the dual‐chamber cohort (*P* = .850).

### 3.3 DX ICD system performance

**Figure 4:**
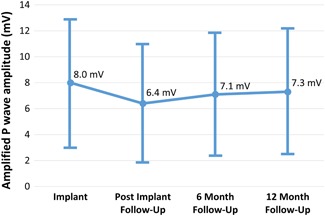
Line plot of amplified sensed atrial amplitudes measured using the DX system over time. Error bars denote standard deviation

In the DX cohort, the mean sensed atrial amplitude was 8.0 ± 5.0 mV at implant and 7.3 ± 4.8 mV at 12‐month follow‐up. At final follow‐up, 11 (7%) patients had sensed atrial amplitude <2 mV and 5 (3%) patients had sensed atrial amplitude <1 mV. Mean sensed atrial amplitudes throughout follow‐up are shown in Figure 4. No DX cohort patients underwent addition of an atrial lead for inadequate atrial sensing or for sinus node dysfunction. Among 75 (50%) patients who had defibrillation thresholds (DFT) measured at implant, the median DFT was less than 20 J (IQR: 10‐40). No DX cohort patients underwent lead revision or addition of a defibrillation coil due to elevated DFTs.

The rate of inappropriate AHRE detection in the DX cohort was 13% (9 of 68 total detections). Of these nine, eight were due to electromagnetic interference, and one was due to lead dislodgement. In the dual‐chamber cohort, the rate of inappropriate AHRE detection was 9% (6 of 68 total detections). Of these six, five were due to sinus tachycardia with far‐field ventricular oversensing, and one was due to electromagnetic interference. There was 100% interobserver agreement in AHRE episode adjudication. Rate of inappropriate AHRE detection in the DX cohort and the dual‐chamber cohort were not statistically different (*P* = .41). No patients in the DX cohort had clinical AF that was undetected by ICD diagnostics.

### 3.4 ICD device therapies

In the DX cohort, 11 (7.3%) patients received 39 episodes of anti‐tachycardia pacing therapy and 3 (2%) patients received three ICD shocks. There were no inappropriate ICD therapies, and all shocks successfully converted ventricular tachycardia. In the dual‐chamber cohort, 15 (10%) patients received 46 ICD shocks at 12 months. Inappropriate therapy data were not available for the dual‐chamber cohort. In the single‐chamber cohort, 10 (6.7%) patients received 21 ICD shocks, and 5 (3.3%) patients received 14 inappropriate ICD shocks.

### 3.5 Device‐related adverse events

In the DX cohort, 8 adverse device‐related events occurred: 2 right ventricular (RV) perforations requiring pericardiocentesis, 2 pneumothorax cases, 2 device infections, and 2 RV lead revisions. There were no device‐related cases of mortality. In the dual‐chamber cohort, 4 adverse events occurred: 1 RV perforation, 2 device infections, and 1 RV lead revision. In the single‐chamber cohort, 9 adverse events occurred: 1 pneumothorax, 2 device infections, 3 RV lead revisions, 1 hematoma evacuation, and 2 deep venous thromboses.

## 4 DISCUSSION

In this multicenter trial, we demonstrate the utility of the DX single‐lead ICD system with an atrial sensing dipole for the detection of AHREs in patients with no prior history of AF. The rate of subclinical AF detection in the DX cohort was comparable to that of a dual‐chamber cohort and higher than clinical AF detection in a single‐chamber cohort. Atrial sensing amplitudes in the DX cohort were stable over time with acceptable rates of AHRE detection accuracy. These findings support a role for the single lead DX system for detection of subclinical AF in ICD patients who do not require atrial pacing.

Subclinical AF is common, as up to 35% of patients with cardiac implantable electronic devices are diagnosed with asymptomatic AF at 30 months.^15^ High‐risk ICD subgroups, such as those with hypertrophic cardiomyopathy, have been found to have a newly detected subclinical AF rate of 50% during long‐term follow‐up.^16^ Most importantly, AHREs have been associated with increased mortality and thromboembolism.^11^, ^12^, ^13^ Furthermore, subclinical AF can cause worsening heart failure, leading to clinical decompensation and hospitalization.^17^, ^18^ Prompt detection of AHREs in patients with ICDs with atrial sensing capabilities can facilitate early treatment of subclinical AF with anticoagulation to prevent strokes and with medical optimization to prevent heart failure exacerbation. Currently, there are no randomized clinical trial data demonstrating the benefit of oral anticoagulation treatment in patients with device‐detected AHREs. The NOAH‐AFNET and ARTESIA studies are currently underway to examine the utility of anticoagulant therapy guided by the presence of device‐detected AHREs.^19^, ^20^

Single‐chamber pacing systems and VDD pacing systems have been associated with a lower rate of procedural complications.^21^ However, the widespread use of early VDD pacing systems has been limited by the reliability of atrial sensing after implant. Only 3% of patients receiving a pacemaker for an atrioventricular block in the United States receive a VDD system and 11% of patients who received a VDD pacing system for AV block had inadequate atrial sensing in long term follow‐up.^22^ The current DX ICD lead has a widely spaced dipole which permits sensing of a larger 49 mm^2^ atrial surface area without the need for direct contact with atrial myocardium.^23^ Furthermore, the atrial signal is amplified four‐fold to allow for adequate atrial sensing, while band‐pass filters and adaptive sensing permit filtering of noise and far‐field R‐waves. In our trial, mean sensed atrial amplitudes in the DX cohort were 8 mV at implant and remained stable at 7 mV at final follow‐up. Only 3% of DX patients had sensed atrial amplitudes <1 mV at follow‐up. Our findings on the atrial sensing performance of the DX system are comparable to those of other studies examining the DX system.^23^, ^24^, ^25^

The accuracy of AHRE detection in the DX cohort was comparable to that of the dual‐chamber cohort despite the increased area of sensing and amplification that could predispose that DX system to oversense external noise. Overall, 87% of AHRE detections in the DX cohort and 91% in the dual‐chamber cohort were true positives, while 13% of AHRE detections in the DX cohort and 9% in the dual‐chamber cohort were false positives. While electromagnetic interference accounted for the majority of inappropriate AHRE detections in the DX cohort, no instances of far‐field ventricular oversensing leading to AHRE detection were seen. Notably, the proportion of false‐positive AHRE detections in the DX cohort in our trial was comparable to the 17% false‐positive AHRE detections reported in the Asymptomatic Atrial Fibrillation and Stroke Evaluation Trial.^26^

Currently, there is conflicting evidence as to whether dual‐chamber discrimination algorithms perform better than single‐chamber discriminators in preventing inappropriate shocks. The OPTION trial showed that dual‐chamber sensing reduced the rate of inappropriate ICD shocks from 10.3% in the single‐chamber setting group to 4.3% in the dual‐chamber setting group.^27^ Another study compared inappropriate therapy rates between the DX system and standard single lead systems and found a significantly lower rate of inappropriate therapies in the DX group (1%) compared to the single‐chamber group (9%).^28^ In our trial, no inappropriate ICD therapies were seen in the DX cohort. While this may have been due to modern ICD programming strategies that utilize higher rate cutoffs and longer detections times, the presence of dual‐chamber discrimination in the DX ICD system may have contributed to this finding.

Despite findings from numerous studies showing an increased rate of procedural complications, in‐hospital mortality, need for lead revision, and need for ICD generator change among patients undergoing dual‐chamber ICD implantation compared to patients undergoing single‐chamber ICD implantation, the rates of dual‐chamber ICD implantation among patients with no atrial pacing indication remain high.^1^, ^3^, ^4^ Our trial shows that the DX ICD system can provide atrial sensing capabilities while maintaining the ease of implant and complication rates of a single lead system. In our study, the adverse device‐related event rates were similar between the DX cohort and the single‐chamber cohort. Other studies have also demonstrated comparable procedural and fluoroscopy times with implantation of DX systems and conventional single‐chamber ICDs.^23^, ^24^, ^25^ One major limitation of the DX system is that it does not offer atrial pacing. However, data have shown that only 3% to 5% of patients implanted with a single‐chamber ventricular ICD require an atrial lead upgrade for sinus node dysfunction during long term follow‐up.^29^ Furthermore, in the DX cohort, no patient required an upgrade to a dual lead ICD system to provide atrial pacing. Therefore, our trial points to an important place for DX ICD system implantations in patients who do not have an indication for atrial pacing but are at risk for the development of subclinical AF.

### 4.1 Study limitations

The major limitation of this trial is the use of historical‐control single‐chamber and dual‐chamber cohorts. As such, there were significant baseline differences in the clinical characteristics between the three device cohorts, which may have affected rates of subclinical AF occurrence. Of note, the CHA_2_DS_2_‐VASc scores of the DX cohort were significantly lower than those of the single and dual‐chamber cohorts, which would potentially suggest a lower baseline risk of AF. Therefore, there was unlikely to be a significant bias towards increased AHRE detection in the DX cohort based on differences in baseline clinical characteristics alone. Furthermore, while patients in the single‐chamber cohort had at least 1 year of follow‐up, which included routine device clinic follow‐up and in‐office ECGs, they were not prospectively monitored for AF. Therefore, there may have been patient‐to‐patient variability in assessment for AF in the single‐chamber cohort. In addition, single‐chamber ICD systems using R‐R interval variability to detect subclinical AF were not available during the study period. Therefore, more contemporary single‐chamber devices using these algorithms could have increased AF detection rates. Finally, this trial was not adequately powered to show statistical non‐inferiority in AHRE detection between the DX cohort and the dual‐chamber cohort.

## 5 CONCLUSION

In this multicenter, prospective cohort‐controlled trial, the use of a single lead DX ICD system with an atrial sensing dipole led to rates of subclinical AF detection that were higher than that of a single‐chamber ICD system and comparable to that of a dual‐chamber ICD system. Atrial sensing characteristics and accuracy of AHRE detections were favorable using the DX system. The DX ICD system may offer significant benefits for AHRE detection in ICD patients who do not have an atrial pacing indication but are at high risk of developing subclinical AF.
